# Recent advances in the *Giardia*–host relationship reveal danger lurking behind the smile

**DOI:** 10.1371/journal.pntd.0006625

**Published:** 2018-09-06

**Authors:** Camila H. Coelho, Steven M. Singer

**Affiliations:** 1 Department of Biology, Georgetown University, Washington, DC, United States of America; 2 Laboratory of Malaria Immunology and Vaccinology, National Institute of Allergy and Infectious Diseases, National Institutes of Health, Rockville, Maryland, United States of America; Hitit University, Faculty of Medicine, TURKEY

**Fig 1:**
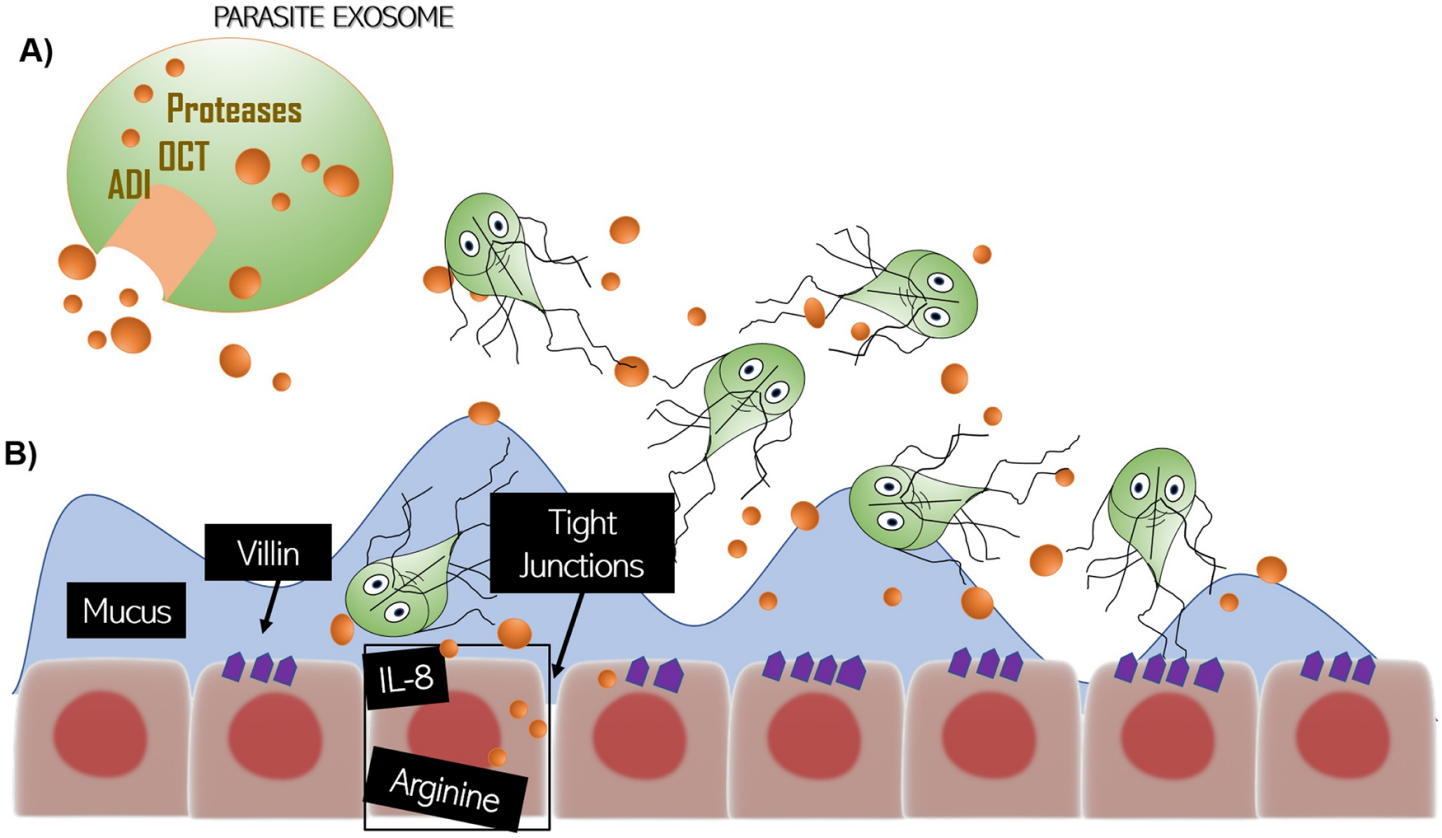
Interaction of *Giardia* and the host. (A) Parasites release proteins, like secreted proteins and proteins contained in macrovesicles and microvesicles, such as ADI, OCT, and several proteases. (B) These secreted factors can degrade tight junction proteins and villin in the epithelial cells, degrade mucins and cytokines secreted by cells, and metabolize arginine resulting in immune evasion and immunomodulation. ADI, arginine deiminase; OCT, ornithine carbamoyltransferase.

Infectious diseases are better understood when virulence factors associated with host interactions are identified and characterized. In giardiasis, several molecules associated to virulence have been identified [1–3]. These include the variant-specific surface proteins that mediate immune evasion [4], a lectin that may contribute to mucosal attachment [5], an arginine deiminase that reduces nitric oxide production by host cells [6], and proteases that degrade cytokines and mucins made by host cells [7, 8] (Fig 1). However, how much variation in these factors exists among *Giardia* strains and how these contribute to the different clinical outcomes of infection are unknown. In addition, full characterization of their interaction with host cells and mechanisms of secretion from the parasite remain lacking.

Two recent studies provide a valuable characterization of host–parasite interactions in giardiasis, delineating the parasite secretome and its interaction with intestinal cells. These studies both use proteomic analysis of proteins secreted by the parasite to assess the interactome of *Giardia* trophozoites with intestinal epithelial cells in vitro. The first study, from the Svärd group, was published in *PLOS Neglected Tropical Diseases* [9] and identified parasite proteins released by the parasite in axenic culture as well as upon exposure to host cells. They further evaluated gene expression, cell signaling, and cytokine production by host cells after exposure to parasite secretory products. The authors analyzed proteins secreted from two *Giardia* strains, GS (Assemblage B) and WB (Assemblage A), commonly used in studies of the parasite and prototypes for the two assemblages most often found in human infections. The number of identified proteins released by WB was greater than those released by GS, either when analyzing axenic cultures or in coculture with human intestinal CaCo2 cells.

The second study, from Dubourg and colleagues, also used proteomics to investigate the secretome of the same two strains, WB and GS [10]. This group used a different human intestinal epithelial cell line (HT-29) to ascertain the effects of secreted factors on host cell physiology. In addition to conventional virulence factors, they suggest that tenascins are involved in producing epithelial damage and disruption of intercellular tight junctions, leading to apoptosis. We previously reported the presence of tenascin-like proteins in the proteome of a virulent zoonotic strain belonging to Assemblage A [11], and tenascins are also among the proteins in the secretome identified by Ma’ayeh and colleagues [9].

The real value of these studies is the comprehensive analysis of the types of proteins secreted, their relative amounts, and their impacts on host cells. The two most abundant proteins secreted into the medium after interaction with intestinal cells, for both GS and WB, were arginine deiminase (ADI) and ornithine carbamoyltransferase (OCT). These enzymes cooperate in catabolizing arginine and producing ATP in the parasite cytosol. ADI has also been suggested to deplete arginine and limit nitric oxide production in the host as a mechanism of immune evasion [6, 12] as well as potentially regulating antigenic variation [4, 13].

Ma’ayeh and colleagues also highlight the importance of the nonconventional secretory pathways present in *Giardia*. Several abundant molecules in the parasite secretome, e.g., ADI, EF-1α, and enolase, lack secretion signal peptides. Ma’ayeh and colleagues identified two types of vesicles ranging in size from 100 to 250 nm in the supernatant of parasite cultures, confirming a recent report of microvesicle release from the parasite [14]. However, it remains to be seen if these vesicles contain the nonsignal peptide-containing proteins. The work from Ma’ayeh and colleagues further addressed the impact of the secreted molecules on host cell signaling and modulation of immune responses.

The work from Ma’ayeh, along with other recent studies, suggests that several candidate virulence factors whose distribution between symptomatic and subclinical infections might be highly informative. In addition to ADI and OCT, Ma’ayeh and colleagues report that both GS and WB release 13 different cathepsin B proteases, and Ortega-Pierres and colleagues [15] have recently demonstrated that a specific *Giardia* protease from the WB strain (named by the authors as giardiapain-1) can induce apoptosis in rat intestinal epithelial cells (IEC-6) and Madin Darby Canine Kidney (MDCK) epithelial cells and degrade proteins at tight junctions. The same group has shown that WB trophozoites constitutively expressing a variant-specific surface protein that has cysteine protease activity (VSP9B10A) induced damage in IEC-6 or MDCK cell monolayers. Similarly, Buret’s group showed in recent studies that proteases from the Assemblage A parasite strain NF can induce cleavage of the cytoskeletal protein villin in CaCo2 cells [16] and degrade mucin produced by LS174T colonic epithelial cells [8]. Proteases from strains NF, WB, and GS could all degrade IL-8 released by host cells [7, 8]. Further investigation is necessary to understand how the secretion system in *Giardia* impacts intestinal tissue in vivo, whether only some or all the proteases secreted by *Giardia* contribute to the symptomatology of the disease and whether interstrain variation in any of these virulence factors contributes to the different clinical outcomes observed in patients.

The *Giardia* secretome studies mentioned above lay the groundwork for new investigations crucial to understanding pathogenesis in *Giardia*. Numerous studies have examined the association between parasite assemblage and virulence in humans. However, the genotyping approaches used to define assemblages rely on sequences of housekeeping genes, usually 18S rRNA, β-giardin, glutamate dehydrogenase, and/or triosephosphate isomerase. What is lacking in the literature is how virulence factors are distributed among parasite assemblages and individual isolates. Both GS and WB were isolated from patients with symptomatic infections, and GS has been shown to induce symptoms in human volunteers [17]. There is no a priori reason to assume that virulence factors are found only in one assemblage but not the other or that the housekeeping genes currently used for genotyping impart virulence to the parasite. Forward genetic approaches are needed to analyze differences in putative virulence gene alleles and expression in isolates from symptomatic and asymptomatic humans. Reverse genetic approaches in cell culture like those used by Cabrera-Licona [18] and in symptomatic animal models, such as gerbils, will also help validate those factors contributing to virulence in vivo. Overexpression of genes in *Giardia* has been possible for 20 years, although strategies for knocking down or knocking out genes in this parasite are much more difficult. Host responses play a pivotal role in pathogenesis in giardiasis as well, as hypermotility and sucrase deficiency are both reduced in immunodeficient animals, and studies of the intersection between parasite virulence factors and host immune response would also be of interest. Finally, the small intestine is home to a robust microbiota, and several studies suggest that interactions among *Giardia*, the host, and these additional microbes all contribute to the eventual outcome of *Giardia* infection [19, 20].
